# Comparison of the mid-term outcomes of banded and non-banded sleeve gastrectomy: safety, food tolerance, and weight regain

**DOI:** 10.1007/s00464-022-09395-4

**Published:** 2022-06-28

**Authors:** Mohamed Hany, Ahmed Sabry, Bart Torensma, Khaled Ahmed, Mostafa Refaie, Ahmed Zidan, Ann Samy Shafiq Agayby, Mohamed Ibrahim, Mohamed Mourad

**Affiliations:** 1 Department of Surgery, Medical Research Institute, Alexandria University, 165 Horreya Avenue, Hadara, 21561 Alexandria Egypt; 2 Depatment of Surgery, Faculty of Medicine, Alexandria University, Alexandria, Egypt; 3 Consultant of Bariatric Surgery at Madina Women’s Hospital (IFSO Center of Excellence), Alexandria, Egypt; 4 Clinical Epidemiologist, Leiden University Medical Center (LUMC), Leiden, The Netherlands

**Keywords:** Banded- and non-banded sleeve, Food tolerance, Weight regain, Safety, Bariatric surgery

## Abstract

**Background:**

Long-term weight regain (WR) after sleeve gastrectomy (SG) is a major challenge. Laparoscopic banded SG (BSG) was introduced to overcome pouch dilation and, consequently, WR; however, its mid-and long-term outcomes have not been sufficiently demonstrated.

**Objective:**

This study retrospectively evaluated the mid-term weight loss efficacy and morbidity over at least a 4-year follow-up after laparoscopic banded SG using a MiniMizer Gastric Ring® and laparoscopic non-banded SG.

**Method:**

The data of 1586 bariatric surgeries were retrospectively evaluated. To ensure homogeneity in our study cohort, propensity score matching (PSM) was performed.

**Results:**

The final cohort comprised 1392 patients: the non-banded SG (*n* = 1260) and BSG (*n* = 132) groups. In our matched cohort (SG, *n* = 655 and BSG, *n* = 132), WR was noted in 4 (3.0%) and 71 (10.8%) patients in the BSG and SG groups, respectively. Gastric band erosion or slippage was not noted in the BSG cohort. The levels of cholesterol and triglyceride were similar in the two groups. Postoperative glycemic control was significantly reduced in the BSG group.

**Conclusion:**

Although the percentage of weight loss achieved in the BSG group was low in the first year postoperatively, the mid-term (sustained) weight loss associated with BSG was superior to that associated with non-banded SG. BSG is a safe procedure with no significant mid-term band-related morbidity; its impact on the resolution of comorbidities is equivalent and perhaps superior to SG.

Sleeve gastrectomy (SG) is one of the most common bariatric procedures performed nowadays [1]. SG is popular because of its safety, simplicity, low postoperative morbidity, and ability to be converted [2]. Despite its proven efficacy, long-term weight regain (WR) after SG is one of the major disadvantages of SG. The percentage of excess weight loss (%EWL) decreased significantly at 5 years postoperatively [3]. In a meta-analysis of nine cohort studies, the recidivism rate was 14–37% after 7 years of follow-up; the authors of this meta-analysis defined recidivism as obtaining a % EWL of < 50% after having an initial %EWL of > 50% [4]. The theories for WR include non-compliance to post SG regimen by the patients and anatomical factors such as gastric pouch dilation [4].

The silastic ring was used to prevent gastric pouch dilation in patients that underwent Roux-en-Y gastric bypass (RYGB) and banded SG (BSG) [5–7]. In 2008, Greenstein and Jacobs applied the gastric band in post SG cases with insufficient weight loss and dilated gastric pouch. The resulting weight loss was encouraging [8]. In the same year, Arceo-Olaiz et al. used the synthetic band during laparoscopic Roux-en-Y gastric bypass with equivalent weight-loss rates after 2 years [9].

Long-term morbidity was the main complication associated with laparoscopic adjustable gastric banding (LAGB) [10]. Complications such as gastric band erosion, oral intolerance, band slippage, gut obstruction, and WR have been reported [11]. After that, LAGB lost its popularity due to poor long-term outcomes, especially insufficient weight loss (IWL) [5].

Laparoscopic BSG was introduced to overcome the pouch dilation and, consequently, WR; however, the mid-and long-term outcomes of BSG on its mid or long-term impact on food tolerance by patients have not been sufficiently reported. Therefore, this study aims to assess retrospectively (over a 4-year follow-up period) the mid-term effects of laparoscopic BSG on weight loss and co-morbidities resolution and food tolerance using a MiniMizer Gastric Ring® (Bariatric Solutions International, Switzerland) and compare them to those of laparoscopic non-banded SG.

## Materials and methods

### Study design

This study was a retrospective cohort study comparing the perioperative and mid-term outcomes of banded and non-banded SG. The study was approved by the Institutional Review Board and conformed to the precepts of the 1975 Declaration of Helsinki.

### Study population

The study population consisted of adult patients who underwent laparoscopic banded or non-banded SG for extreme obesity at the Medical Research Institute Hospital and Alexandria University Main Hospital between January 2016 and December 2017.

### Eligibility for bariatric or metabolic surgery

The surgical indication was extreme [body mass index (BMI) > 40 kg/m^2^] or severe (BMI 35–40 kg/m^2^) obesity with comorbidities interfering with the quality of life, after evaluation by a multidisciplinary team, according to the National Institutes of Health recommendations [12, 13]. All the patients scheduled for SG were offered the option of having BSG or SG, and the decision was made by the patients after the advantages and disadvantages of the procedures were explained, including the cost, unavailability of long-term outcomes, and psychological effects. All the patients provided informed consent to undergo BSG or SG and anonymously use their data for research.

### Surgical technique

SG was performed as previously described [6]. Two teams performed the SGs, and all the BSGs were performed by the same surgeon in a multi-disciplinary team setting. Approximately 70–80% of the gastric volume was resected using a 40 French bougie. For BSG, perigastric dissection was performed 4–5 cm from the gastroesophageal junction, and a size 7.5 (1.75 cm internal diameter) MiniMizer Gastric Ring® (Bariatric Solutions International, Switzerland) was placed loosely around the pouch.

Non-absorbable sutures were used to fix the ring to the stomach passing through the built-in holes in the ring. Concomitant operative procedures included crural repair when hiatal hernia was present, using unidirectional barbed 2/0 V-Loc non-absorbable sutures (Covidien, Mansfield, MA, USA), and cholecystectomy using the same ports without adding extra ports.

### Follow-up after surgery

All the patients were regularly followed-up at the outpatient clinic over four years (at 6 months and 1-, 2-, 3-, and 4-years postoperatively). The following data were collected at each visit: the body weight, BMI, postoperative hemoglobin, fasting plasma glucose, cholesterol, triglycerides levels, serum albumin, ferritin, calcium, vitamins D3 and B12, and renal and liver functions. Routine endoscopy was performed for all the patients 1 year postoperatively. Additional endoscopies were performed depending on the patients’ symptoms.

### Data retrieval from records and definitions

The data were retrieved, and the preoperative baseline characteristics were collected, including the age, sex, body weight, height, BMI, operation time, symptoms of gastroesophageal reflux disease (GERD), endoscopic findings of GERD, hiatal hernia, and gallstones detected by ultrasonography and comorbidities (including diabetes mellitus, hypercholesterolemia, hyperlipidemia, ischemic heart disease, essential hypertension, impaired renal function, obstructive sleep apnea, and history of upper abdominal surgery).

Data on BMI, EWL, total weight loss (TWL), WR, amelioration of comorbidities, and serial laboratory investigations, including HbA1c, fasting blood glucose level, hemoglobin, calcium, vitamin D3, and vitamin B12, were obtained at each visit.

Food tolerance was assessed for all the patients at the first and fourth-year follow-up visits using a one-page questionnaire (with the scores ranging between 1 and 27) with questions on overall alimentation satisfaction, meal timing through the day, and several food types tolerability, and vomiting and regurgitation events. The higher the score, the better the food tolerance [14].

The mid-term morbidity was assessed using postoperative symptoms of GERD, food intolerance, constriction at the incisura or band site, and band erosion or slippage. Additionally, the conversion rate to other bariatric surgeries due to IWL, WR, or mid-term complications was determined.

The percentage total weight loss and %EWL were calculated using the formulae: (weight loss/the initial weight) and (weight loss/baseline excess weight) × 100, respectively, where weight loss = preoperative weight − initial weight × 100, baseline excess weight = initial weight − ideal weight (X), and X = 23 kg/m^2^. X was calculated using an ideal BMI (23 kg/m^2^) [15] IWL = EWL of < 50%. [16] Insufficient weight loss was defined as EWL < 50% after 1 year from surgery [17]. WR was defined as 10% regain of the nadir weight at the last follow-up visit [18, 19].

Endoscopic grading for reflux esophagitis was done using the Los Angeles Classification of Gastroesophageal Reflux. The Dindo-Clavien score was used to assess the 30-day-postoperative morbidity [20]. A score of ≥ 3 indicates severe postoperative morbidity.

### Statistical analysis

Continuous variables were presented as median and interquartile ranges and compared using the Mann–Whitney *U* test, whereas categorical variables were presented as numeric proportions and compared using the χ2 test, Fisher’s exact test, or Monte Carlo test [21].

To minimize the differences in the baseline characteristics of the patients between the groups, propensity score matching (PSM) was performed. The covariates included in the propensity score were age, sex, BMI, essential hypertension, diabetes mellitus, obstructive sleep apnea, dyslipidemia, osteoarthritis, preoperative gastroesophageal reflux, presence of hiatal hernia, gallstones, cardiac disease, psychological disorders, thrombo-vascular complications, neoplasm, alcohol intake, and smoking status. The matching algorithm was used according to the nearest neighbor method, with a 1:5 ratio (without replacement) and a caliper width of 0.2. The balance between the two groups was assessed using the standardized mean difference for baseline characteristics [22].

The outcome variables were compared in the two matched groups using the logistic regression analysis with a robust variance estimator. Repeated measure analysis of variance was used to measure variable changes. The mid-term outcome was assessed by calculating the time to successful weight loss using the Kaplan–Meier method, and a comparison was performed using the stratified log-rank test. Univariable logistic regression analysis was performed to identify the impact of BSG (successful EWL and WR) compared with SG. All the calculations were performed using IBM Corp. Released 2017. IBM SPSS Statistics for Windows, Version 25.0. Armonk, NY: IBM Corp, and R software version 3.3.3 (R Foundation for statistical computing). All the tests were two-tailed, and statistical significance was established at *p* < 0.05. Graphs were drawn using GraphPad Prism 8.0.1 software.

## Results

### Patient demographics

**Fig. 1:**
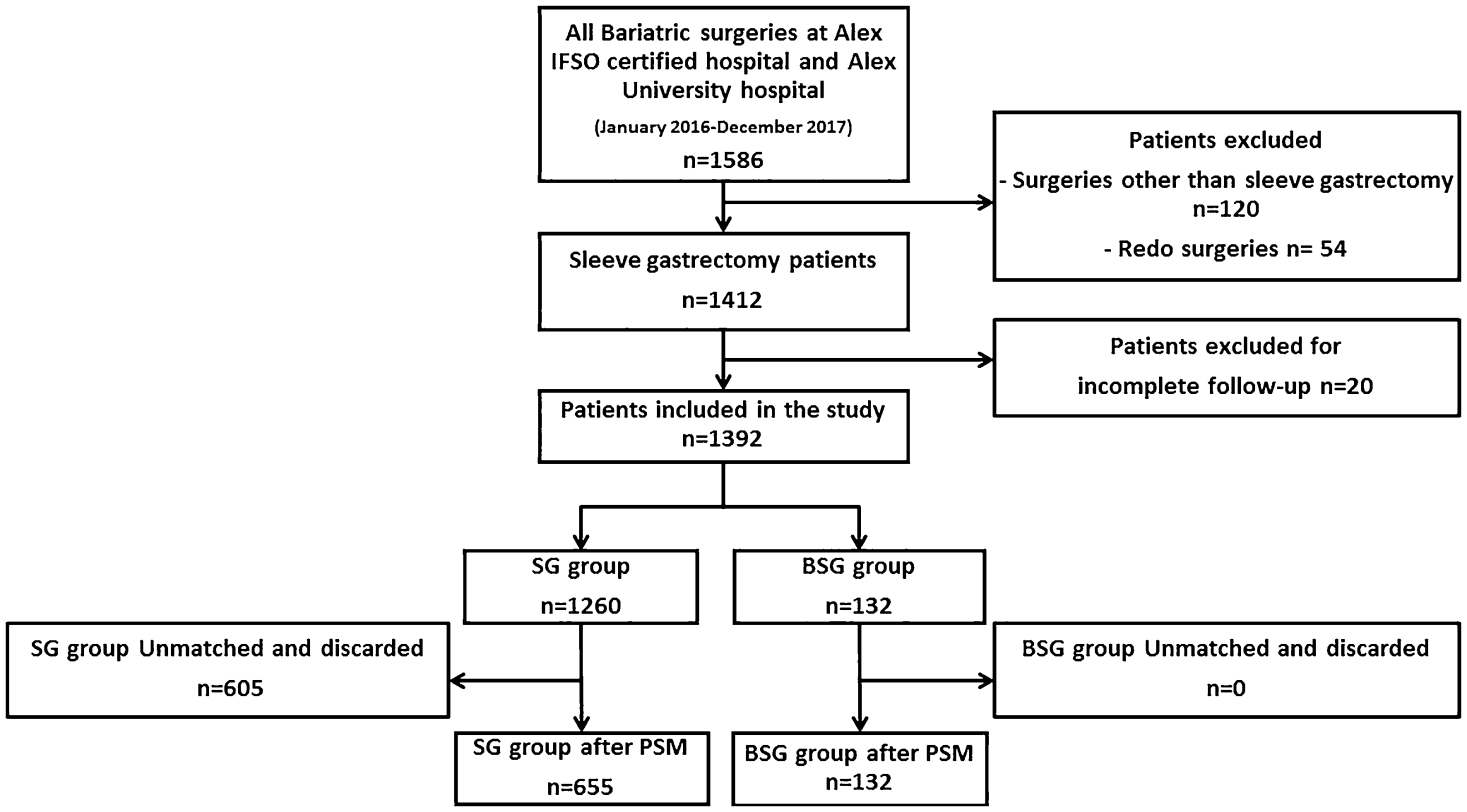
Study flow chart. *BSG* banded sleeve gastrectomy, *IFSO* International Federation for the Surgery of Obesity and Metabolic Disorders, *PSM* propensity score matching, *SG* non-banded sleeve gastrectomy

The data of 1586 bariatric surgeries performed between January 2016 and December 2017 at the Medical Research Institute Hospital and Alexandria University Main Hospital were collected. Furthermore, 174 patients who underwent surgeries other than SG and redo surgeries were excluded. Twenty patients with incomplete follow-up records were excluded. The final cohort comprised 1392 patients who were divided into the non-banded SG (*n* = 1260) and BSG groups (*n* = 132) (Fig. 1).

The baseline characteristics were compared between the two groups. No significant difference was noted in the demographic features (such as age and sex) and anthropometric measures (including waist circumference, height, weight, and BMI) between the groups. Moreover, the preoperative comorbidities, including essential hypertension, diabetes mellitus, obstructive sleep apnea, dyslipidemia, osteoarthritis, preoperative gastroesophageal reflux, the presence of hiatal hernia, gallstones, menstrual abnormality, cardiac diseases, psychological disorders, thrombo-vascular complications, neoplasm, alcohol intake, or smoking, were similar in the groups. None of the patients in our cohort had positive serology markers for hepatitis B or C (Table 1).

**Table 1:** Baseline characteristics before propensity score matching

	SG^a^ *N* = 1260	BSG^b^ *N* = 132	*p* value
*Demography*			
Sex (M/F)	321/939	28/104	0.282
Age (years)	34.0 (28.0–42.0)	34.5 (27.0–40.0)	0.492
*Comorbidities*			
HTN^c^	381 (30.2%)	40 (30.3%)	0.988
DM^d^	218 (17.3%)	24 (18.2%)	0.800
OSA^e^	160 (12.7%)	19 (14.4%)	0.580
Dyslipidemia	379 (30.1%)	40 (30.3%)	0.957
Osteoarthritis	322 (25.6%)	34 (25.8%)	0.960
Cardiac disease	80 (6.3%)	8 (6.1%)	0.897
Psychological disorders	162 (12.9%)	16 (12.1%)	0.810
Vascular diseases	191 (15.2%)	20 (15.2%)	0.998
DVT^f^, PE^g^	20 (1.6%)	2 (1.5%)	1.000*
Neoplasm	23 (1.8%)	2 (1.5%)	1.000*
Gallstones	129 (10.2%)	13 (9.8%)	1.000
Smoking	445 (35.3%)	47 (35.6%)	0.947
Alcohol	18 (1.4%)	2 (1.5%)	1.000*
*Preoperative endoscopic assessment*			
Hiatal hernia	78 (6.2%)	7 (5.3%)	0.685
Esophagitis	254 (20.2%)	25 (18.9%)	0.739
*Anthropometric measures*			
Waist (cm)	115.0 (108.0–132.0)	116.0 (108.0–132.0)	0.902
Height (m)	1.67 (1.60–1.74)	1.67 (1.59–1.74)	0.874
Weight (kg)	130.0 (115.0–146.0)	130.0 (115.0–145.9)	0.916
BMI^h^	47.5 (42.7–52.1)	47.4 (42.8–52.3)	0.985
*Parameters of glycemic control*			
FBS^i^	96 (85–108)	96 (86–106)	0.844
HbA1cj	8.0 (7.0–9.0)	8.0 (7.0–9.0)	0.958

Categorical variables are expressed in counts (percentages). Continuous variables are expressed in median values (interquartile range)

^a^Variables used in the propensity score matching

^b^Fisher’s exact test

^a^*BG* Sleeve gastrectomy,

^b^*BSG* Banded sleeve gastrectomy

^c^*HTN* essential hypertension

^d^*DM* diabetes mellitus

^e^*OSA* obstructive sleep apnea

^f^*DVT* deep vein thrombosis

^g^*PE*: ^h^*BMI*: body mass index

^i^*FBS* fasting blood sugar

^j^*HbA1c* glycated hemoglobin, *HCV* hepatitis C virus, *PS* propensity score

To ensure homogeneity in our study cohort, PSM was performed. After PSM, 605 patients in the SG group were unmatched and excluded from the study cohort, and the remaining 658 patients and all the patients in the BSG group were matched and included in the study.

After that, all the baseline characteristics of the matched groups were compared. All the variables were equally distributed in the study groups, with no significant differences. In addition, the standardized mean difference (SMD) was < 0.1 for all the variables (Table 2).

**Table 2:** Baseline characteristics after propensity score matching

	SG^a^ group (*n* = 655)	BSG^b^ group (*n* = 132)	*p*	SMD^c^ Before	SMD After
*Demography*					
Sex M/F^a^	135/523	28/104	0.857	− 0.104	0.030
Age (year)^a^	30.0 (24.0–39.0)	34.5 (27.0–40.0)	0.821	− 0.111	0.028
*Comorbidities*					
HTN^d, a^	190 (28.9%)	40 (30.3%)	0.753	0.001	0.026
DM^e, a^	113 (17.3%)	24 (18.2%)	0.797	0.023	0.020
OSA^f, a^	93 (14.1%)	19 (14.4%)	0.938	0.048	0.034
Dyslipidemia^a^	202 (30.7%)	40 (30.3%)	0.928	0.005	0.021
Osteoarthritis^a^	163 (24.8%)	34 (25.8%)	0.811	0.005	0.078
Cardiac disease^a^	46 (7.0%)	8 (6.1%)	0.699	− 0.012	− 0.013
Psychological disorders^a^	82 (12.5%)	16 (12.1%)	0.914	− 0.022	0.018
Vascular diseases^a^	106 (16.1%)	20 (15.2%)	0.784	< 0.001	− 0.007
Thromboembolic complication (DVT^g^, PE^h^)^a^	11 (1.7%)	2 (1.5%)	1.000^b^	− 0.006	− 0.025
Neoplasm^a^	14 (2.1%)	2 (1.5%)	1.000^b^	− 0.025	0.012
Gallstones^a^	59 (9.0%)	13 (9.8%)	0.742	− 0.013	0.030
Smoking^a^	214 (32.5%)	47 (35.6%)	0.492	0.006	− 0.020
Alcohol^a^	11 (1.7%)	2 (1.5%)	1.000^b^	0.007	− 0.025
*Preoperative endoscopic assessment*					
Esophagitis^a^	130 (19.8%)	25 (18.9%)	0.829	− 0.031	0.020
Hiatal hernia^a^	35 (5.3%)	7 (5.3%)	0.991	− 0.039	0.007
*Anthropometric measures*					
Waist	115.0 (108.0–131.3)	116.0 (108.0–132.0)	0.882	0.016	0.011
Height ‘m’	1.7 (1.6–1.7)	1.7 (1.6–1.7)	0.950	− 0.010	0.003
Weight ‘kg’	129 (113–146)	130 (115–146)	0.816	0.001	0.019
BMI^i a^	47.2 (42.5–52.1)	47.4 (42.8–52.3)	0.725	0.008	0.025
*Parameters of glycemic control*					
FBS^j^	96.0 (86.0–108.0)	96.0 (85.0–108.0)	0.757	0.010	0.019
HbA1c^k^	8.0 (7.0–9.0)	8.0 (7.0–9.0)	0.945	− 0.001	− 0.019

Categorical variables are expressed in counts (percentages). Continuous variables are expressed in median values (interquartile range)

^a^Variables used in the propensity score matching

^b^Fisher’s exact test

^a^*SG* Sleeve gastrectomy ^b^*BSG* Banded sleeve gastrectomy ^c^*SMD* standardized mean difference, ^d^*HTN* essential hypertension, ^e^*DM* diabetes mellitus, ^f^*OSA* obstructive sleep apnea, ^g^*DVT* deep vein thrombosis, ^h^*PE*: ^i^*BMI*: body mass index, ^j^*FBS* fasting blood sugar, ^k^*HbA1c* glycated hemoglobin, *HCV* hepatitis C virus, *PS* propensity score

The operative time was equivalent in both cohorts (Table 3). The three cases of gastric leakage were managed by stent insertion with favorable outcomes. The 30-day-postoperative severe morbidity rates were comparable in the study groups (*p* = 1.0). GERD was encountered in 109 patients (16.6%) in the SG group vs. 19 patients (14.4%) in the BSG group, with no significant difference (*p* = 0.552). Furthermore, 111 patients (16.8%) in the SG group required conversion to RYGB. For the indications for conversion were weight regain in 51 (7.75%), GERD in 43 (6.5%), and GERD with WR in 17 (2.6%) patients. No patient needed conversion in the BSG group throughout the 4-year follow-up period (*p* < 0.0001). It is noteworthy that in the BSG group, 25 patients presented with esophagitis preoperatively, and the postoperative routine endoscopy in 21 of them (84%) showed regression of the reflux.

**Table 3:** Comparison of operative and postoperative data between both the study groups

	SG^a^ group (*n* = 655)	BSG^b^ group (*n* = 132)	*p*
Operative time (min)^†^	41.63 ± 7.45	41.82 ± 7.55	0.766
Concomitant cholecystectomy	59 (9.0%)	14 (10.6%)	0.621
Concomitant hiatal hernia repair	34 (5.2%)	7 (5.3%)	1.000
Postoperative leakage	3 (0.5%)	0 (0%)	1.000^a^
30-day severe postoperative morbidity	3 (0.5%)	1 (0.8%)	1.000^a^
*Postoperative endoscopic findings*			0.514^b^
Normal endoscopy	503 (76.8%)	103 (78.0%)	
GERD^c^ grade B*	109 (16.6%)	19 (14.4%)	0.552
Hiatal hernia	30 (4.6%)	7 (5.3%)	0.763
Constriction at the incisura angularis	13 (2.0%)	–	
Constriction at the ring	–	3 (2.3%)	
Conversion to RYGB	111 (16.9%)	0 (0%)	< 0.0001

Categorical variables are expressed in counts (percentages). Continuous variables are expressed in median values (interquartile range)

^a^Fisher’s exact test

^b^Monte Carlo test

^a^*SG* Sleeve gastrectomy, ^b^*BSG* Banded sleeve gastrectomy

^†^Operative time represents the main surgery without additional procedures

^*^^c^GERD was assessed using endoscopic Los Angeles classification

No case of gastric band erosion or slippage was noted in our BSG cohort. Three patients (2.3%) in the BSG group experienced solid dysphagia and reflux symptoms during the follow-up period. Endoscopy revealed constriction at the band site that required a few endoscopic pneumatic balloon dilation sessions with satisfactory results.

Regarding the impact on the comorbidities, at the 4-year follow-up visit, the cholesterol and triglyceride levels in the SG and BSG groups were equivalent with *p* = 0.713 and 0.969, respectively. In addition, the fasting blood sugar (FBS) levels were not significantly different between the study groups (Table 4).

**Table 4:** Postoperative laboratory work-up and impact on comorbidities

	SG^a^ group (*n* = 655)	BSG^b^ group (*n* = 132)	*p*
Hb^c^	12.5 (11.0–14.0)	12.5 (11.0–14.0)	0.834
Cholesterol	207 (170–260)	209 (170–260)	0.713
Triglycerides	178 (150–240)	178 (150–243)	0.969
Albumin	4.0 (3.6–4.6)	4.0 (3.6–4.6)	0.805
Ferritin	112 (79–187)	109 (76.5–175.5)	0.466
Vitamin D3	29 (24–38.2)	29 (25–38)	0.986
Vitamin B12	325 (214–532)	325 (214–536)	0.650
FBS^d^	95.0 (83.0–106.0)	96.0 (83.0–104.0)	0.847

Categorical variables are expressed in counts (percentages). Continuous variables are expressed in median values (interquartile range)

^a^*SG* Sleeve gastrectomy

^b^*BSG* Banded sleeve gastrectomy

^c^*Hb* hemoglobin: ^d^*FBS* fasting blood sugar

**Fig. 2:**
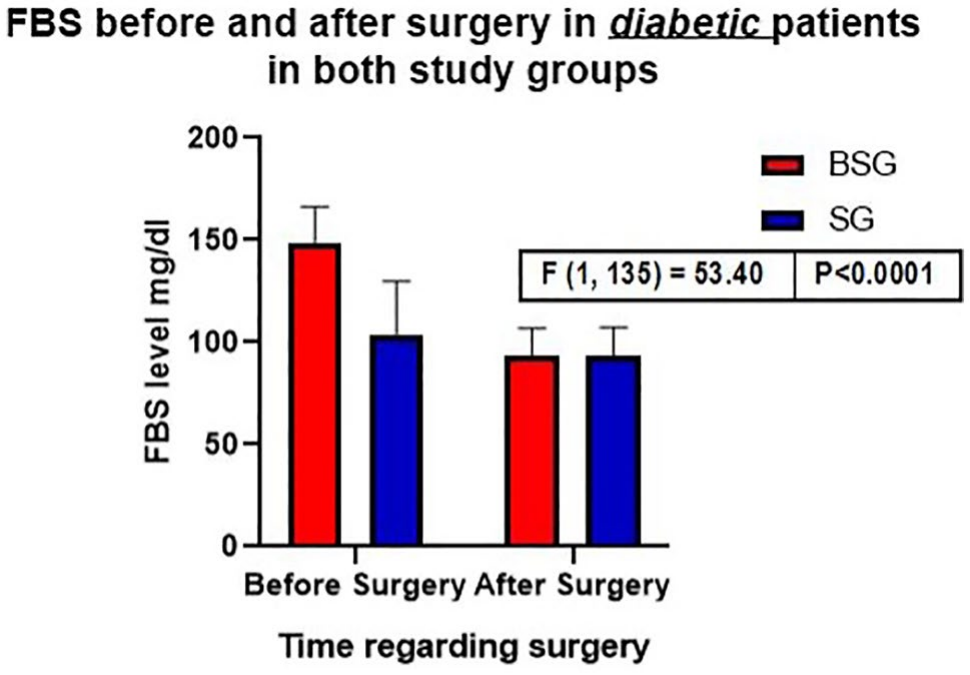
Bar chart comparing the impact of FBS in patients with diabetes before and after surgery in each study group

Analysis of the 137 patients with the comorbidity diabetes in our matched cohorts showed that although the patients in the BSG group had a higher mean preoperative FBS level (149 mg/dl versus 104 mg/dl in the BSG and SG groups, (*p* < 0.001), the postoperative glycemic control was equivalent between the groups (postoperative FBS level of 94 mg/dl in the groups,

(*p* = 0.995), but significant difference before and after surgery in both groups. (*p* < 0.0001) (Fig. 2).

**Fig. 3:**
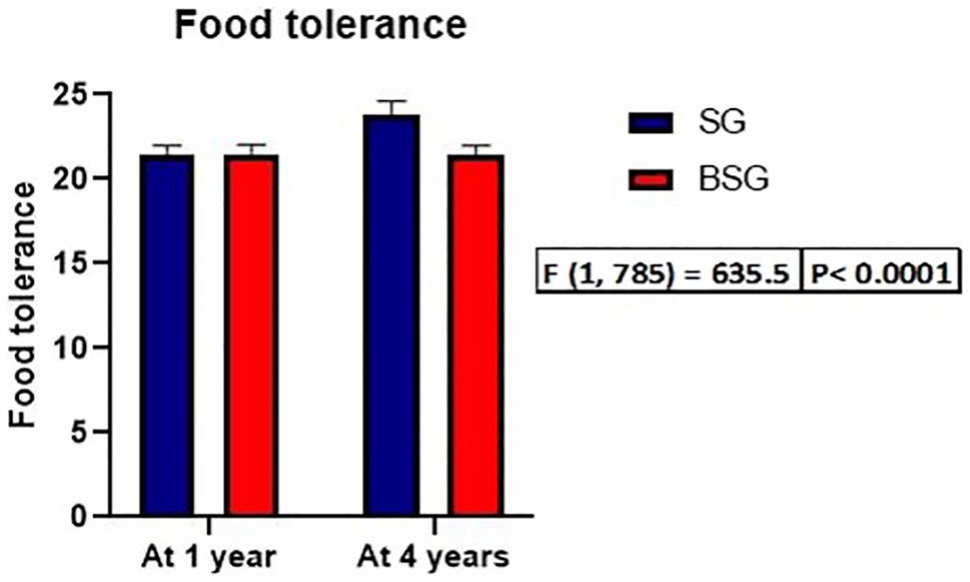
Bar chart comparing the food tolerance at 1 and 4 years after surgery in each group

The median score for food tolerance after 1 year postoperatively of the groups was the same (21). On the contrary, the median score for food tolerance at the end of the 4th year postoperatively was 24 for the SG group. It was significantly higher than that of the BSG group, which remained unchanged at 21 (*p* < 0.0001) (Fig. 3). During the index procedure, a concomitant cholecystectomy was performed 59 (9.0%) and 14 (10.6%) times, and concomitant hiatal hernia repair was performed 34 (5.2%) and 7 (5.3%) in SG and BSG groups, respectively (Table 3).

### Matched cohort

In the matched cohort, 4 cases (3.0%) of WR were noted in the BSG group and 71 (10.8%) in the SG group, with a significant difference (*p* < 0.001). At the 1-, 2-, 3- and 4-year follow-up visits, all the patients in the BSG group successful reached the EWL of > 50%, whereas the %IWL rates in the SG group were 3.1%, 2.3%, 3.7%, and 5.3%, respectively, with no significant difference between the groups. No single case in the BSG group has reached a nadir weight reflecting insufficient weight loss (< 50% EWL). Meanwhile, nine patients in the SG group have reached nadir reflecting insufficient weight loss, only one had WR, while the remaining eight patients did not.

The rate of successful weight loss (EWL ≥ 50%) at 6 months was higher in the SG group than in the BSG group and gradually increased till 4 years postoperatively. Meanwhile, all the patients in the BSG group had reached successful weight loss at the 1-year follow-up visit (*p* = 0.003).

**Fig. 4:**
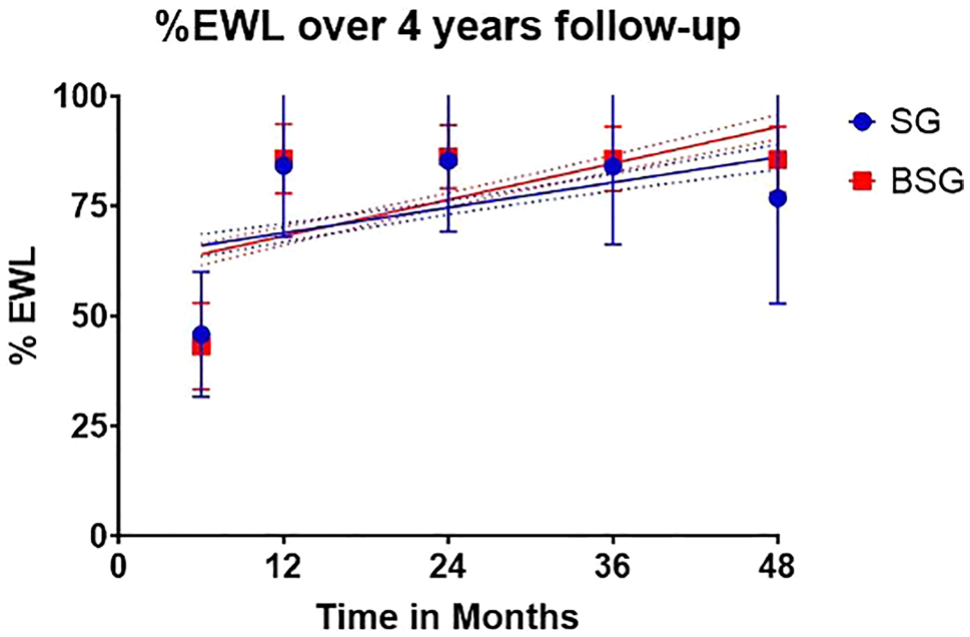
Graph comparing EWL% between the groups over 4 years of follow-up

The EWL% at 6 months was significantly lower in the BSG group than in the SG group (*p* = 0.037), the EWL% rates were not significantly different between the study groups at the 1-, 2-, and 3-year follow-up visits (Fig. 4).

## Discussion

This is one of the largest studies to evaluate and compare the outcomes of BSG with non-BSG over a mid-term follow-up period of 4 years in all the included patients. In addition, this study cohort comprised two identical study groups with comparable baseline characteristics using PSM. We achieved an SMD of < 0.1 for all the baseline variables in the groups.

Laparoscopic SG was initially introduced as a step in a two-stage bariatric surgery in patients with extreme obesity to minimize the surgical risk. The short-term outcomes of SG, in terms of EWL, resolution of comorbidities, and safety, with low rates of postoperative complications, are excellent [3]. The EWL% was 86% at 1 year postoperatively. There was marked resolution of hypertension, obstructive sleep apnea (99%), and diabetes mellitus (957%) in > 90% of the patients [3]. After restrictive surgeries, including SG, long-term (sustained) weight control is still a challenge [23–25].

In our study, WR at 4 years postoperatively was 3.0% in the BSG group and 10.8% in the SG group (*p* < 0.001). This finding supports the suggested role of BSG in overcoming the disadvantage of non-banded SG (that is, sustained EWL rates). Moreover, at the end of the first year postoperatively, 100% of the patients in the BSG group had achieved successful weight loss, which was maintained till the end of the 4-year follow-up period. Meanwhile, in the non-banded SG group, an increasing trend in IWL was noted starting from the end of the second postoperative year. This explained the importance of the ring in preventing gastric dilation, which is believed to be responsible for WR following non-banded SG. The other possible mechanism includes the additive role of gastric banding in activating the peripheral satiety pathway [26].

The initial %EWL at 6 months of follow-up in this study was statistically significantly lower in the BSG cohort than the SG cohort, but unclear if this has clinical relevance. We do not have a clear explanation for this issue. Still, many causes have been reported for impaired weight loss after bariatric surgery, including behavioral problems, physical inactivity, and hormonal factors [17]. The overall weight loss after surgery throughout the follow-up in this study is consistent with data published in the literature [25, 27–34]. The most logical thoughts are that a band can be positioned as an “extra” weight loss product, but more as an “anti”-weight regain product. The sleeve operation is primarily the reason why patients lose weight with the 70–80% gastric volume restriction of the stomach and that the ring will help after sufficient weight loss that the regain will be prevented. The literature shows equal initial weight loss between the BSG and the SG or better weight loss in the BSG [25, 27–31, 33, 34]. However, data from a randomized prospective study has shown an initial lower %EBMIL in the BSG cohort compared to the SG cohort [32].

The food tolerance score in our study can explain the difference in weight loss, and weight regain between the study groups at different periods. The food tolerance was similar at the end of the first year in the groups, and this correlated with the equivalent %EWL at the same time. Meanwhile, food tolerance significantly increased at the end of the 4th year postoperatively in the SG group compared to a stable food tolerance in the BSG group at the same time, which correlated with the significantly higher rate of WR in the SG group at 4-year postoperatively than in the BSG group.

The incidence of vomiting after BSG was higher in the first 12 months when the meal volume and pouch dilatation proximal to the band increased [27]. We could not demonstrate similar findings regarding the incidence of vomiting. Food tolerance is still unfavorable among the patients that underwent BSG.

Although Alvarenga et al. have reported a %EWL of 86% at 1-year post SG, this rate dropped to 63% 5 years postoperatively. This was partially attributed to the drop-out rate and the incomplete follow-up of some of their study participants [3]. Furthermore, Himpens et al. reported a low %EWL of 53% in the sixth-year post SG [24]. Bhandari et al. recently reported that the absolute weight loss was significantly higher at the start of the second year postoperatively in the BSG group than in the SG group [28]. Even though previous studies reported equivalent change in BMI at 6 and 12 months postoperatively, in our study, BSG was associated with a significantly lower EWL than non-banded SG (43% versus 45%; *p* = 0.037) at 6 months postoperatively. Unlike other previous studies, our study showed similar operative time and length of hospital stay for the SG and BSG groups [28].

Regarding comorbidities, fasting blood glucose, serum cholesterol, and triglyceride levels were similar postoperatively in the study groups. In addition, patients with diabetes in the BSG group showed a more significant drop in their FBS level than those in the SG group. This refutes the previous unexplained findings (in the literature) of higher HbA1c and FBS levels in the BSG group than in the SG group [28]. Fink et al. have explained the reduction in the symptoms of regurgitation and reflux in patients in the BSG group by the reflux barrier effect of the ring [29]. This could explain the high rate of esophagitis remission after BSG in this study.

Three patients (2.3%) presented with ring site strictures during the postoperative follow-up visit. Those patients presented with solid dysphagia and persistent reflux symptoms, and the endoscopy revealed strictures at the site of the ring, which was not tight and was passable to the scope in all cases. We tried Pneumatic balloon dilation before considering the removal of the minimizer ring. We performed the dilatation using 20 mm pneumatic balloons inflated for one minute twice in the same session. The three patients responded well to the dilatation sessions and had improvement in their symptoms. The reason for the improvement of symptoms may be an improvement in the adaptation of the stomach with dilatation or even psychological relief. Some authors reported management of stenosis after BSG by increasing the band length to 7.5 cm or removal of the band due to dysphagia or severe reflux symptoms [6, 27, 30]. We apply the ring loosely around the gastric sleeve pouch at 7.5 cm in length from the start.

No band slippages nor erosions were detected. The occurrence of band erosions in BSG may be an extremely rare complication that was not reported by authors who addressed the BSG [25, 27, 31, 34]. Band slippage after BSG is also a rarely reported complication; it was reported by Fink et al. in one patient who was managed by removal of the band [30].

These results confirm the safety of the technique in the mid-term. Similarly, Gentileschi et al. reported no band-related complications over an extended follow-up period in a smaller cohort [32]. These results may be attributed to the loose application of the ring (compared to the traditional gastric band), leading to fewer chances of gastric erosion and stricture; moreover, the limited dissection of the pars flaccida is responsible for the lower rate of ring slippage.

In this study, the conversion rate from SG to RYGB in the SG cohort was significantly higher than in the BSG cohort, 16.9% vs. 0%, respectively (*p* < 0.001). Conversion of LSG to another bariatric procedure is now a well-reported issue in the literature, ranging from 4.7% to 20.7%, reaching up to 40% in higher volume centers [4, 33]. RYGB is the most reported revision procedure after LSG in the literature (75.2%), followed by resleeve (18.7%) [33]. In our practice, we choose RYGB as a revisional procedure for patients with SG who have symptomatic grade “B” or more reflux esophagitis not responding to medical treatment or WR/insufficient weight loss with uniform dilatation of the gastric sleeve pouch without residual fundus or antrum. We adopt a re-sleeve only when the gastric sleeve pouch has residual fundus or antrum. When the gastric sleeve pouch is uniformly dilated, conversion to RYGB is the best option [4, 33].

The reported rates of revisional surgery after BSG are lower than SG in the literature, ranging from 2 to 5%, with the most common causes of conversion being band slippage and GERD. At the same time, the most performed procedures are removal of the band or increasing the band length and conversion to RYGB [6, 27, 30]. Also, the banded SG is also reported to have lower rates of GERD when compared to LSG [34]. In this study, we had similar findings regarding the incidence of GERD, which may be correlated to the band's presence that mechanically prevents the reflux of the gastric juice into the esophagus while the part of the stomach above the ring has few acid-secreting glands. Also, WR incidence in the BSG cohort was lower than in LSG. The causes of conversion in this study were WR (7.75%), GERD (6.5%), and combined GERD with WR (2.6%). The most-reported indication for conversion after LSG is the weight regain (70%), followed by GERD (16%) [4]. This coincides with our findings.

To the best of our knowledge, our study is one of the largest studies to date to evaluate the mid-term outcome of BSG; however, there are some limitations. First, the postoperative gastric volumetric studies were not included to compare the pouch dilatation between the groups. Second, the analysis of the resolution of the comorbidities in the study groups was incomplete. Third, routine endoscopy was only performed for all patients 1 year after surgery and was repeated later only for patients with symptoms. Some patients may have missed asymptomatic problems like GERD and Hiatal hernias or complications related to the band.

Finally, some of our initial SG participants were discarded after propensity score matching; however, these patients had initially refused to undergo BSG.

## Conclusion

Although the %EWL achieved in the BSG group was low in the first year postoperatively, the mid-term (sustained) weight loss associated with BSG was superior to that associated with non-banded SG. BSG is a safe procedure with no significant mid-term band-related morbidities. The impact of BSG is equivalent and might be superior to that of SG in terms of resolution of comorbidities such as DM and GERD.
